# Supplementary figures and images for: Heterologous expression, purification and structural features of native Dictyostelium discoideum dye-decolorizing peroxidase bound to a natively incorporated heme
Source: Front Chem. 2023 Aug 1;11:1220543. doi: 10.3389/fchem.2023.1220543 (PMC10427876; doi:10.3389/fchem.2023.1220543)

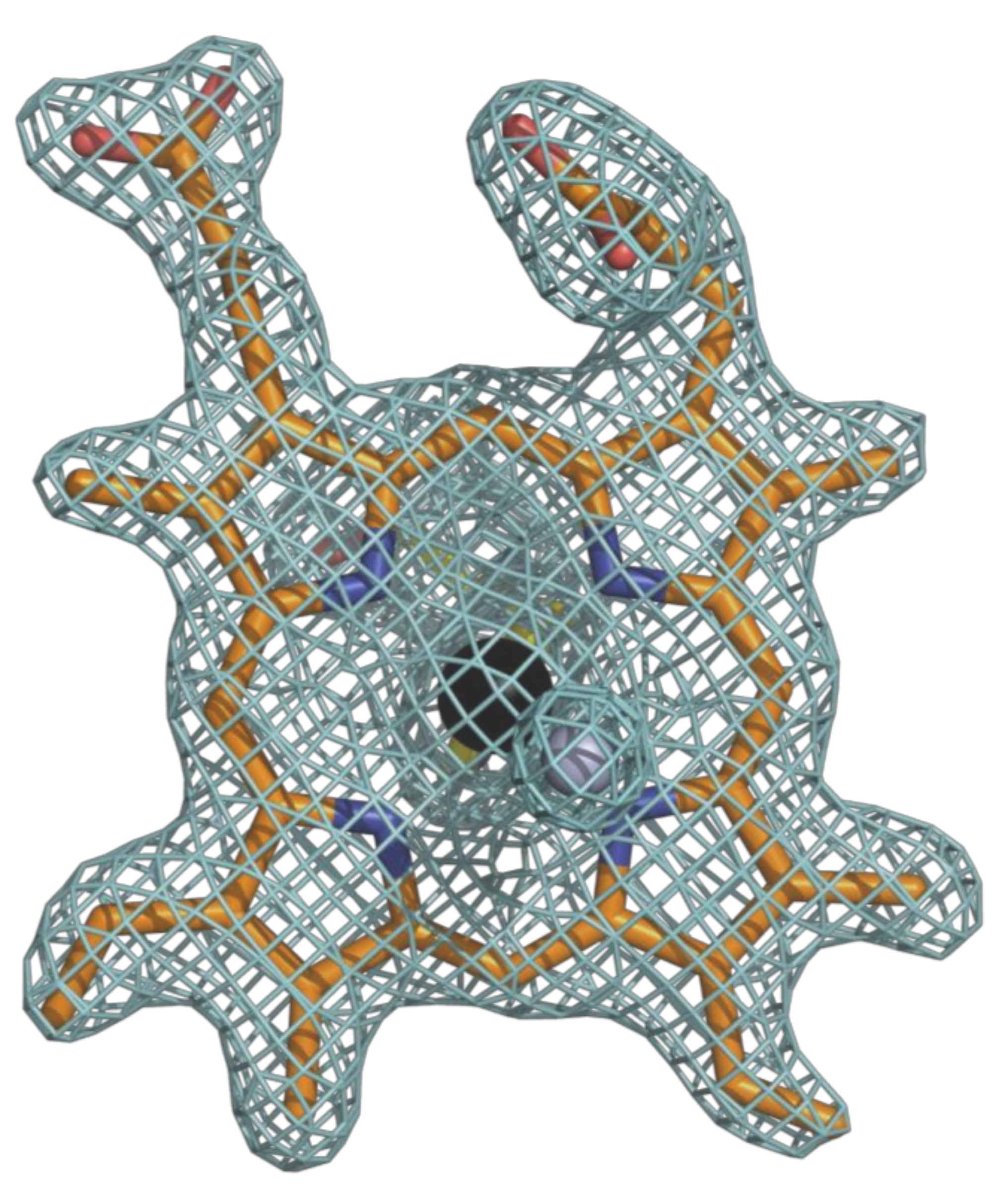

Supplement: Supplementary file 1 [file Image2.TIF]

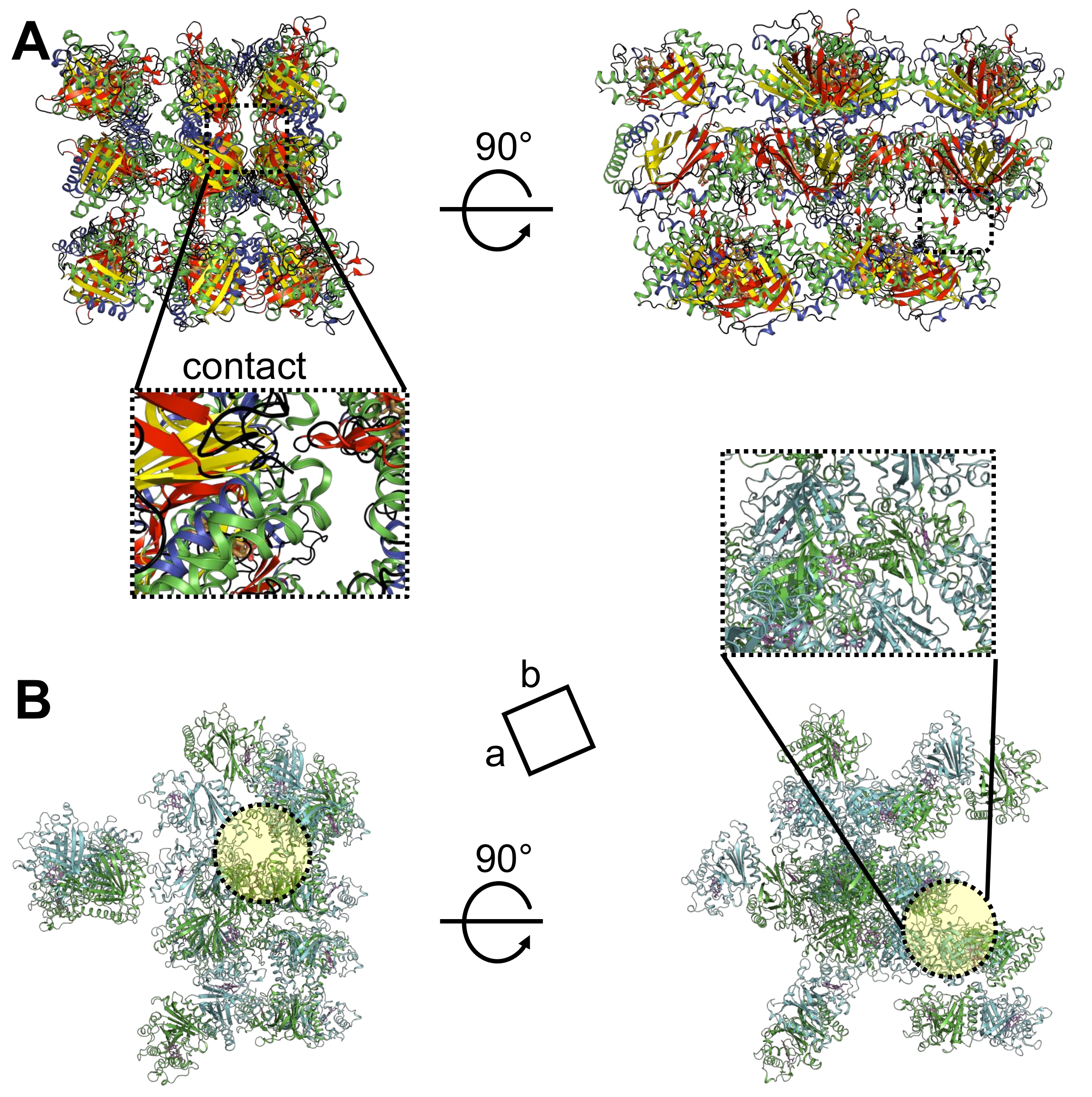

Supplement: Supplementary file 2 [file Image1.TIF]

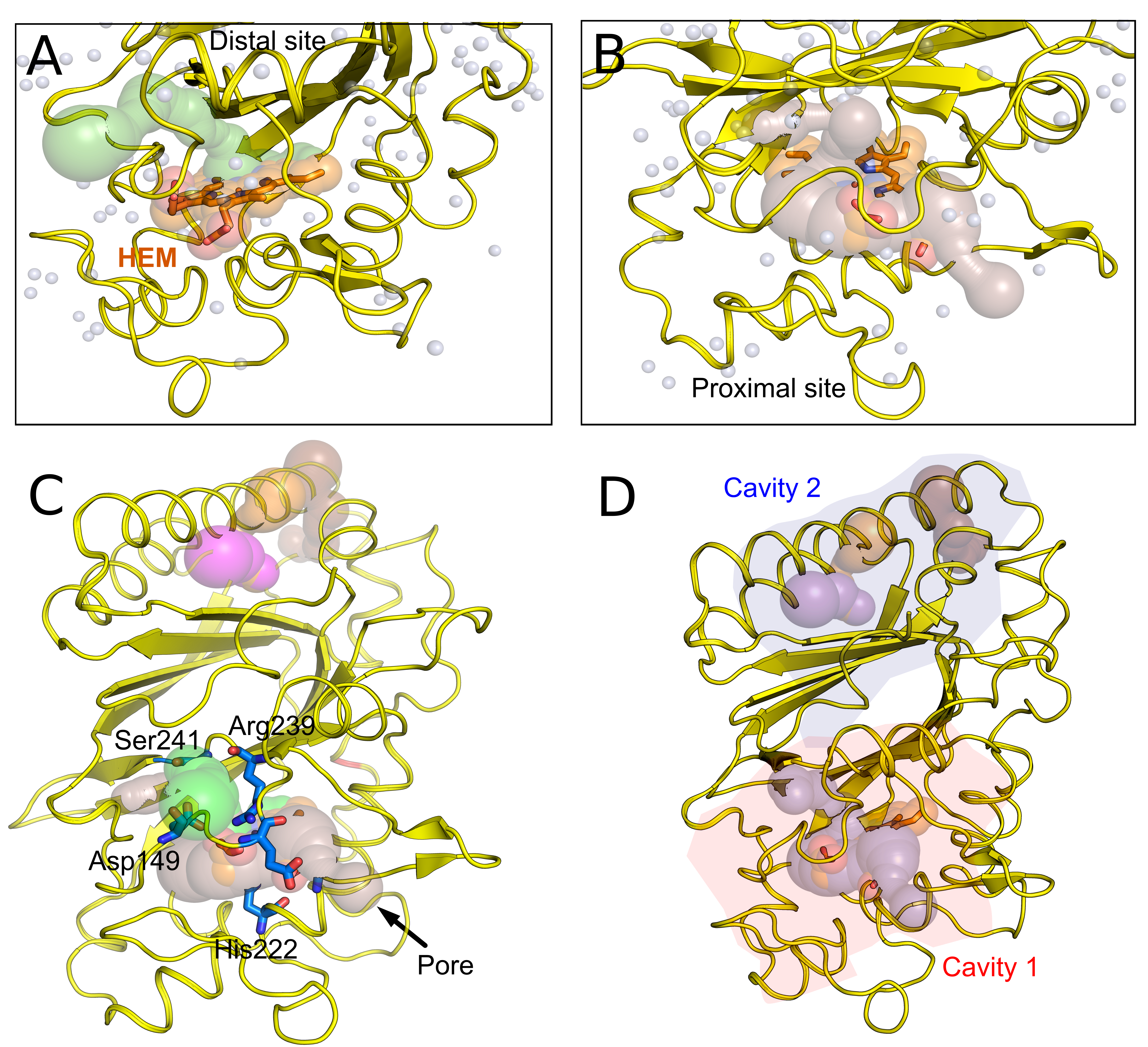

Supplement: Supplementary file 3 [file Image3.PNG]
